# Molding of the Superior Maxilla in Adenoid Vegetations

**Published:** 1897-06

**Authors:** 


### Molding of the Superior Maxilla in Adenoid Vegetations.

According to *Rev. Hebdom. de Laryng., d'Otol., et de Rhin.*, Korner, in 1891, drew attention to the particular change of form which takes place in the upper jaw, at two different periods. When adenoids obstruct the nares during the first dentition, elevation of the palatine vault is produced, along with a sudden retardation of development in the whole upper jaw; the transverse diameter is shortened, and the longitudinal lengthened.

After the Second dentition, the following changes are added to the above: The bony palate becomes still more elevated; the alveolar borders approximate still closer, and the maxilla seems to be compressed laterally, producing an angle at the median line; the two middle incisors turn their lingual surfaces toward each other, and a transverse section of the maxilla assumes an oval form. In nasal obstructions, of other origin, the secondary changes of the maxilla are not like these—the angle of the median line and the V-position of the middle incisors being exclusively found with adenoid vegetations.

These changes are denied to rickets, because those due to rickets are found in parts of the body in full development. This occurs in the cranium in the first years of life, whereas the molding of the superior maxilla from adenoids occurs most often after the second dentition. Besides, rachitic anomalies occur only on the *lower* jaw, while nasal obstruction changes due to adenoids are limited to the *upper*.

Finally, Rosenberg, in 1895, pointed out the frequent absence or decadence of the *lateral* incisors in these cases. Reyntjes has seen a case of elevated palate without adenoids. Bloch found elevated palate related to modification of the lateral cavities; but, along with Sikkel, maintains that the median line angularity presents only with adenoids.

"WOULDN'T it be terrible, Robbie," said little Mabel, as they drank their morning's milk, "if there wasn't no cows?" "Yeth," said Robbie, "we'd have to dwink condensed milk then, and it's horrid."

